# The effects of surface topography modification on hydrogel properties

**DOI:** 10.1063/5.0046076

**Published:** 2021-07-27

**Authors:** Linan Cui, Yuan Yao, Evelyn K. F. Yim

**Affiliations:** 1 Department of Chemical Engineering, University of Waterloo, Waterloo, Ontario N2L 3G1, Canada; 2 Waterloo Institute for Nanotechnology, University of Waterloo, Waterloo, Ontario N2L 3G1, Canada; 3 Centre for Biotechnology and Bioengineering, University of Waterloo, Waterloo, Ontario N2L 3G1, Canada

## Abstract

Hydrogel has been an attractive biomaterial for tissue engineering, drug delivery, wound healing, and contact lens materials, due to its outstanding properties, including high water content, transparency, biocompatibility, tissue mechanical matching, and low toxicity. As hydrogel commonly possesses high surface hydrophilicity, chemical modifications have been applied to achieve the optimal surface properties to improve the performance of hydrogels for specific applications. Ideally, the effects of surface modifications would be stable, and the modification would not affect the inherent hydrogel properties. In recent years, a new type of surface modification has been discovered to be able to alter hydrogel properties by physically patterning the hydrogel surfaces with topographies. Such physical patterning methods can also affect hydrogel surface chemical properties, such as protein adsorption, microbial adhesion, and cell response. This review will first summarize the works on developing hydrogel surface patterning methods. The influence of surface topography on interfacial energy and the subsequent effects on protein adsorption, microbial, and cell interactions with patterned hydrogel, with specific examples in biomedical applications, will be discussed. Finally, current problems and future challenges on topographical modification of hydrogels will also be discussed.

## I. INTRODUCTION

Hydrogel is defined as a three-dimensional (3D) network of polymer chains that can swell and retain a significant fraction of water inside its structure without dissolving in water. Its hydrophilic properties mainly come from the hydrophilic functional groups, while the interactions between the network polymer chains protect it from dissolving in water.^1,2^ Due to the high water content, hydrogel has good biocompatibility, tunable biodegradability, and low toxicity, making it an ideal material for biological and medical applications both *in vivo* and *in vitro.*^3,4^ For example, poly(ethylene glycol) (PEG) hydrogels have been used in controlled drug release;^5^ polyvinyl alcohol (PVA) hydrogels are used for contact lens, wound dressing, and artificial cartilage, and recently showed promise in vascular implanting and tissue-mimicking;^6^ gelatin methacryloyl (GelMA) hydrogels are suitable for fabricating functional bone scaffolds and biosensing;^7^ and silicone hydrogels have been developed mainly as contact lens materials.^8^

Despite the various advantages, the hydrogel has some common problems, such as unexpected bacteria adhesion,^9^ undesired protein adsorption,^10^ and lack of mechanical strength,^11^ which are limiting its applications. To solve such problems and enhance the functions of hydrogel, surface modifications are frequently performed to improve the surface properties. Technologies have been developed to enhance surface properties of the hydrogel, including chemical, biochemical, and topographical modification.

In recent years, many studies have illustrated that the incorporation of surface topographies can alter material surface properties, such as hydrophilicity, surface energy, and cell interactions,^12–15^ without affecting the bulk properties of the substrate material. This gave inspiration to the modification of hydrogels that their surface properties can also be changed via different surface topographies.

This review will discuss the effects of topographical modifications on hydrogel material properties, including hydrophobicity, protein deposition, bacteria adhesion, cell responses, and mechanical properties based on previous research. A summary of current topographical modification techniques will be provided. Also, the challenges for future development will be discussed.

## II. TECHNIQUES TO FABRICATE PATTERNED HYDROGELS

Surface construction methods can be divided into two types depending on the final surface topographical conditions. The first category is the surface roughening method. Surface roughening methods aim to change the surface roughness and are usually applied to metallic or plastic materials. Surface roughness refers to the height or depth of asperities and irregularities on the surface in both macro- and microscales. The most commonly used parameters describing the roughness are average surface roughness (R_a_) and root mean square surface roughness (R_rms_), which can be calculated from the average and root mean square deviation of height values from the surface mean line, respectively. Examples of roughening methods include surface silanization,^16^ Taguchi design,^17^ and severe shot peening.^18^

**FIG. 1.**
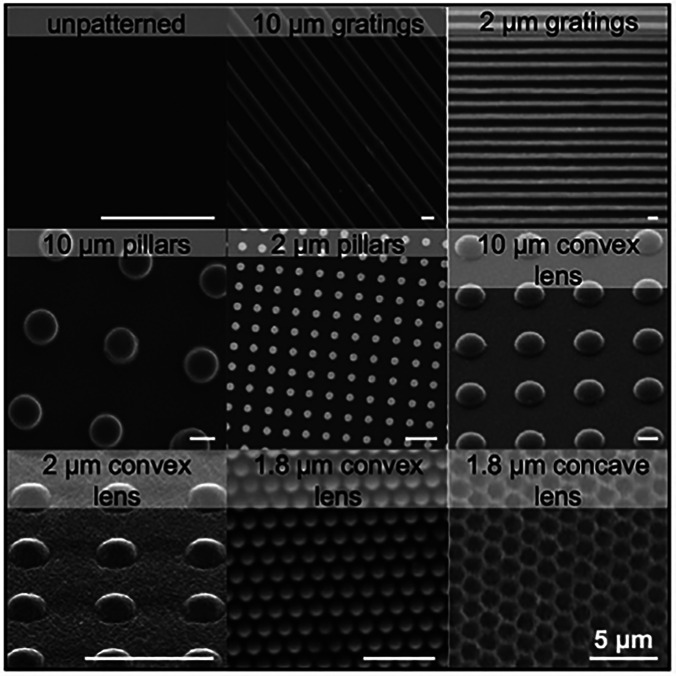
Examples of predesigned patterns with different shapes and sizes on hydrogel surfaces. Reprinted with permission from Cutiongco *et al.*, Biomaterials **84**, 184–195 (2016). Copyright 2016 Elsevier.^23^

Different from roughening that mainly creates random and polydisperse surface features, surface patterning methods produce specific micro/nanoscale topographies on material surfaces that are periodic or precisely predesigned (Fig. 1). Based on specific requirements and designs of the material, various patterning methods have also been developed to be applied to different materials, such as soft lithography,^19^ template-based surface nanopatterning,^20^ nanoimprinting,^21^ and direct laser interference patterning.^22^ The selection of methods depends on both the inherent properties of modified materials and the advantages and disadvantages of each method.

Due to the high water content, any change in volume of hydrogel resulting from swelling or deswelling can subsequently cause surface deformation, such as feature widening, making it difficult to precisely obtain the initially designed patterns. Also, extensive swelling can occur in hydrogels with higher precursor concentration, resulting in the undesirable detachment of the hydrogel layer from the substrate during the patterning process.^24,25^ Compared to densely crosslinked stiff hydrogels, loosely crosslinked soft hydrogels are more prone to damage during the demolding step as they could easily break into debris under mechanical stress.^26^ In addition to the fragility of hydrogel, the adsorption of protein-based hydrogel precursor onto templates, such as polydimethylsiloxane (PDMS) without surface treatment, due to the nonspecific protein adsorption onto surfaces, could also affect the demolding process.^24,27,28^ Therefore, it is challenging to apply conventional surface patterning techniques mentioned above to hydrogel materials directly.

The casting method is one of the most commonly used methods for hydrogel patterning. Cross-linking hydrogel solution is usually poured onto the surface of a prepared negative mold with specific patterns, so the precisely predefinable patterned hydrogel can be obtained after demolding the crosslinked hydrogel from the mold.^23,25,29,30^ Another popular way to fabricate surface topographically patterned hydrogel is photolithographic patterning technique, where the mixed solution of photoinitiator and monomer are layered onto the photoactive hydrogel substrate and exposed to UV light through the photolithographic mask with desired patterns.^31,32^ Other commonly used methods, such as nanoimprinting,^23,33–35^ 3D printing,^36–38^ electrospinning,^39–41^ multiphoton patterning,^42–45^ e-beam lithographic patterning,^46,47^ Self-assembly wrinkle technique,^48,49^ ion-induced nanopatterning,^50^ and swelling-induced patterning,^51,52^ also have their own specific fabrication mechanism and process. In addition, many research groups have also developed effective methods to add patterns to hydrogel substrates. For example, dithiol macromolecular linker that can both bond to gold covalently and entangle the PEG hydrogel network was used to transfer a cell-adhesion-available gold microarray from the initial glass substrate to a cell-adhesion-resistant PEG hydrogel surface;^53^ and Peng's group has successfully obtained surface patterned hydrogels via ion inkjet printing.^54^ Features of these techniques and the resolution they can reach have been listed in Table I.

**TABLE I.** Description and comparison between common hydrogel surface patterning methods.

Common surface patterning methods	Description	Feature size range/resolution	Advantages	Disadvantages	References
Casting method	Cross-linking hydrogel solution is poured on top of negative molds	Sub-micrometer/microsize features	• Simplicity	• Mold materials should have good wetting properties	23, 25, 30, and 55
			• Low cost		
			• Versatility	• Potential pattern collapse during demolding due to excessive stress of mold materials	
Thermal-based nanoimprint lithography (for thermo-plastic polymer)	The substrate will be heated up and soften into a molten stage, and it will fill in the negative mold cavities under specific pressure and time.	Nanoscale features (down to 6 nm)	• High resolution used for planar patterning	• Precise temperature control needed	33 and 34
			• High-throughput process	• Temperatures much higher than glass transition temperature T_g_ can cause serious damage to the substrate	
Nanoimprint lithography	Cross-linking of hydrogel on the patterned mold happens during nanoimprinting.	Sub-micrometer-size features	• High resolution	• Limited to materials that can be crosslinked during the nanoimprinting process	23 and 35
			• Simplicity		
Photolithographic patterning	Specific hydrogel regions are exposed to UV light through the transparency mask.	Microsize features (a few micrometers to a few hundred micrometers)	• Simple	• Only large size patterns can be obtained.	31 and 32
			• Inexpensive	• Photomasks necessary	
3D printing	Stimuli-responsive hydrogel is fabricated layer by layer from a 3D model that is generated by computer-aided-design (CAD) software	Microsize features	• Fast	• Lack of various printable hydrogel systems	36–38
			• Inexpensive		
			• 3D structure easily designed by CAD software	• Limited resolution and feature size	
Ion inkjet printing	The cross-linking density of the printed regions will be increased by the complexation between the polyelectrolyte and ferric ions, and the hydrogel can have shape deformation upon swelling/deswelling	Resolution up to several hundred micrometers	• Programmable variation in cross-linking densities	• Patterning strongly relies on the shapes of metal anodes	54
			• Controllable swelling and deswelling behavior of the hydrogel	• Not suitable for continuous or mass production of complicated patterns	
Electrospinning method	The hydrogel is formed by stabilizing the nanofibers (applied either during or after the spinning process) and rapid dissolution is prevented via re-exposure to water	Difficult to achieve pattern with size (diameter or pore) >50 *μ*m	• Low cost	• Limited to random and aligned fibrous structure	39–41
			• High throughput		
			• Tunability		
			• Both the morphology of individual fibers and the topography of the entire nanofibrous scaffold are controllable		
Multiphoton patterning method	Direct laser writing enables patterning of 3D microstructures without photomasks or complex optical systems; photosensitive crosslinker is used in the fabrication	Sub-micrometer /microsize features	• High resolution	• Precise control of laser wavelength needed	42–45
			• Free-form 3D fabrication		
			• Noncontact fabrication	• Limited to photo-crosslinkable materials	
			• High resolution		
e-beam lithographic patterning method	Hydrogel is crosslinked upon exposure of accelerated electrons to create patterns on the substrate	Sub-micrometer/microsize features		• Longer processing time	46 and 47
				• Expensive	
				• Dose tests are always necessary and significant to precisely obtain the expected feature size and shape	
			• Complex patterns can be printed directly		
Swelling-induced surface patterning method	The photocurable hydrogel is exposed to the light in air and then swelling; the anisotropic osmotic pressure in depth makes the outer surface buckled and create the pattern	Tens of micrometers	• Stable patterns in both dry and swollen states	• The control of final pattern morphology could be challenging	51 and 52
			• Simplicity, additional coating or organic solvents are unnecessary for swelling		
Self-assembly wrinkle technique	The pre-polymerized hydrogel is spin-coated and deswelling in vacuum, then exposed to UV to form wrinkled patterns	Microsize features	• Simplicity	• Not suitable for specifically designed patterns	48 and 49
			• Fast		
Ion-induced nanopatterning method	Ions are used in the directed plasma nanosynthesis to create the nanostructures on hydrogel surface	Nanosized features	• Reproducible fabrication	• Not suitable for specifically designed patterns	50
			• Stable to sterilization		
			• Mechanically stable		
			• Nanostructures with high aspect ratio can be fabricated without collapse		

In general, due to the special physical and chemical properties of hydrogel materials, several techniques have been developed from conventional surface construction methods to pattern hydrogels. Based on the hydrogel type and the desired application, these techniques could also vary from each other in detail.

### A. Impact of the patterning technique on surface chemistry

While the changes in chemical moiety will be an important property to characterize for surface modifications, most studies on surface topography patterning focused on the changes in interfacial surface energy with limited characterization on the surface chemistry of hydrogel. Part of the reasons could be attributed to the study design of the surface patterning studies, as most of the studies compared the patterned and unpatterned surfaces fabricated by the same technology,^45,56^ or chemical modification would also be performed on the patterned hydrogel.^57,58^

Looking at the examples of the impact of fabrication method on the surface chemistry of topographically patterned polymers, the impact on surface chemistry could vary with patterning technique or methods, and it will also depend on the polymer or hydrogel materials. Various patterning methods could cause surface chemistry changes. Electrospinning has been shown to alter the fluorine surface concentration of polymethyl methacrylate random tetrahyrdroperflourooctyl acrylate.^59^ Liu *et al.* compared the degree of denaturation of collagen between acetic acid‐spun fibers and 1,1,1,3,3,3 hexafluoroisopropanol‐spun fibers. They demonstrated a lower degree of denaturation in acetic acid-spun fibers, indicating that the solvent used in electrospinning plays a major role in affecting the ultimate surface chemistry of electrospun fibers.^60^ In the process of soft lithography fabrication of polydimethylsiloxane (PDMS), silanization of the master surface is frequently carried out to produce passivated surfaces to prevent irreversible bonding with PDMS.^61,62^ Silanization has been shown to increase surface hydrophobicity. Ion-induced lithography also changes surface chemistry depending on ion beam parameters and the reactivity of ion species. XPS results showed that Ar^+^ and O_2_^+^ irradiation introduces contaminants, such as iron, molybdenum, calcium elements, on the surfaces.^63^ An ultrafast multiphoton laser has also been reported to cause chemical changes in polyimide films.^64^ In addition, many researchers have explored patterning approaches to fabricate surfaces with controlled topographies and surface chemistry. e-beam lithography has been used with plasma treatment to create a chemically patterned surface, suggesting that e-beam lithography could be used to alter surface chemistry.^65^ Similarly, 3D printing has been applied with wet chemical modification to fabricate surfaces with controlled functionality and microstructure.^66^ The combination of lithography with coating also generated surfaces with tunable wettability.^67^

To the knowledge of the authors, a number of studies have examined hydrogel's surface chemistry change after surface patterning and showed that the patterning method showed minimum impact on the surface chemical moiety. For example, the surface elemental composition of PVA hydrogel samples fabricated from casting and thermo-based nanoimprinting was verified by x-ray photoelectron spectroscopy, and no noticeable difference was observed between these samples.^23^

While the primary objective of introducing a topographical pattern would be to alter the interfacial energy, understanding how the patterning fabrication could alter the chemical moiety would be essential to study the surface properties. The limited number of studies with thorough chemical characterization identifies a knowledge gap that researchers should also examine the potential changes in chemical moiety induced by surface patterning in the future.

### B. Stability of topographical features on hydrogel

Hydrogel surface topography is an important modulator of surface properties, and the stability of the topography can critically influence its performance. Stability of patterned features will include (1) the patterning fidelity and the maintenance of the fidelity, for example, if the features could be easily collapsed; (2) the stability of dimensions, for example, if there will be changes of pattern dimension upon rehydration; and (3) the stability of features over time.

**FIG. 2.**
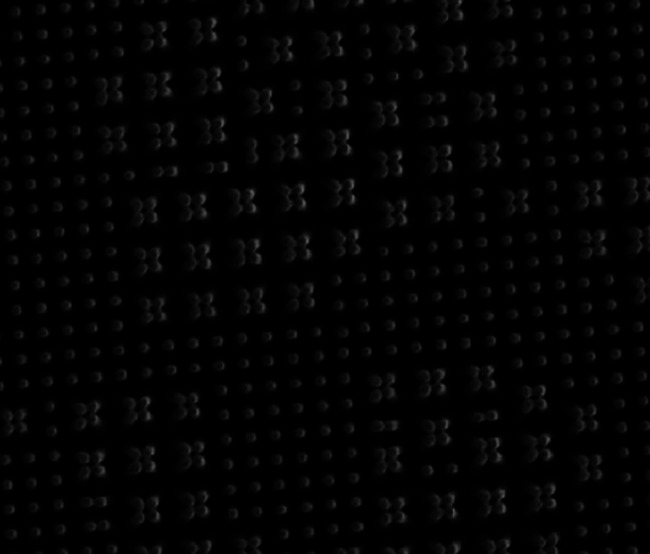
Scanning electron microscopy (SEM) images of poly(2-hydroxylethyl methacrylate)-poly(methyl methacrylate) copolymer (PHEMA-co-PMMA) micropillars clustered due to water capillary force. Reproduced with permission from Chandra and Yang, Acc. Chem. Res. **43**(8), 1080–1091 (2010). Copyright 2010 American Chemical Society.^68^

The pattern fidelity mainly depends on the pattern features and patterning methods. It is generally noted that soft, high-aspect-ratio microstructures, such as high pillars, could buckle under their own weight. When the spacing distance between pillars decreases, collapse can possibly occur that neighboring pillars bend laterally and adhere to each other.^68^ In addition to weight, pillars can also be attracted or repelled from each other due to the capillary force when they are partially immersed in liquid,^69^ as is shown in Fig. 2. Such structure deformation is usually undesirable and should be avoided.

The patterning method is another influential parameter to affect the pattern fidelity on the hydrogel. For example, as one of the most commonly used methods, the casting method can create patterns on the hydrogel surface by demolding the crosslinked hydrogel from the pre-patterned negative molds. However, those microstructures can be easily damaged during the demolding process due to excessive stress. The development of the demolding damage-free method, therefore, draws attention as well.^55^

Hydrogel is known to be able to absorb and retain a large amount of water inside the polymer matrices, and the phenomena of dehydration and rehydration are common during the fabrication process and in various biomedical applications. The swelling or deswelling behaviors occurring in these processes would depend on the swelling ratio of the hydrogel and can also cause feature deformation.^70^ As is introduced above, hydrogel-induced swelling behavior can even be employed as a specific patterning method. The swelling behavior utilized in these methods will be controllable and precisely designed. However, undesirable structure deformation upon dehydration or rehydration could exist and affect the structure dimension. For example, the feature dimensions of the cast PVA hydrogel were measured by scanning electron microscopy (SEM) after dehydration in air, the diameter values of both 10 *μ*m pillars and 10 *μ*m convex lenses were reduced to 6 and 7.5 *μ*m, respectively.^23^ The degree of cross-linking could also determine if hydration will significantly affect the dimensions. The dimensions of the topographical structure on sequential crosslinked GelMA (GelMA+) were measure before and after hydration.^56^ The height and width of the grating pattern were not significantly different upon hydration. However, the characterization of the changes in dimension upon hydration could be technically challenging for sub-micrometer topographical structures. Most of the conventional surface microscopy techniques have limited capacity to characterize the hydrated hydrogel surface with high resolution, and these challenges will be further discussed in Sec. VIII B.

The third factor in determining the stability of hydrogel surface topography is the stability over time. Depending on the specific usage and application, different patterned hydrogels were designed for studies of different durations. Most studies in the literature focused on developing the hydrogel for a specific application to be used within a limited time period or for a short duration. Some studies have designed and developed dynamic, stimuli response topographical-patterned hydrogels, such as photodegradable or photoresponsive hydrogel pattern,^71,72^ thermos-responsive hydrogel pattern,^73–75^ or biodegradable patterns.^56^ Thus, the stability or the responsiveness of the patterned feature could also depend on the properties of the hydrogel, such as the thermal-stability, cross-linking, and biodegradation.

A few papers examined the topographical features directly or indirectly over a period of time. For example, surface patterned PVA hydrogel has been shown to maintain its surface topography for 4 weeks after *in vivo* implantation and after one year in the sterile phosphate-buffered saline (PBS) solution.^23^ However, the stability of patterned hydrogels over time is largely unexplored, which deserves future study.

## III. INFLUENCE OF SURFACE TOPOGRAPHY ON INTERFACIAL ENERGY

### A. Influence of surface topography on the hydrophobicity of hydrogels

**FIG. 3.**
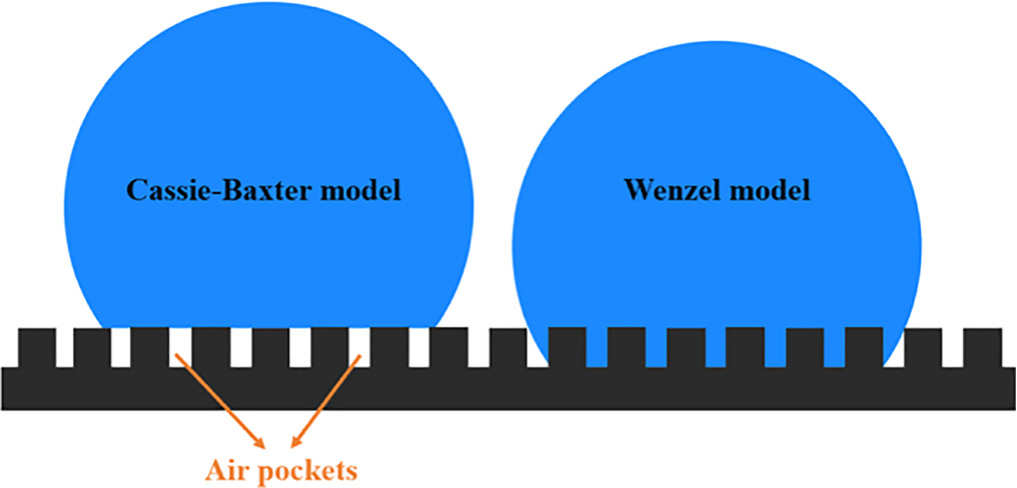
Cassie–Baxter model vs Wenzel model.

In the past few decades, many studies have shown that the wetting state can be changed by adding different surface topographies, in addition to being determined by the intrinsic hydrophobic or hydrophilic properties of the material.^76–80^ Two models, the Cassie–Baxter model and the Wenzel model, have been proposed to describe the process when a droplet is placed on a solid surface. In the Cassie–Baxter model, the droplet will only touch the top of the topography, when air would be trapped between the micrometer-sized asperities. While in the Wenzel model, the microstructures will penetrate the droplet (Fig. 3).^81–83^ Dai *et al.* have identified that the magnitude of the interaction between the droplets and substrates can be varied by the height and width of pillar structures. When the water contact angle on a smooth surface is larger than 93.13°, increasing the height of pillars (2.82 nm width) to 3.76 nm can change the wetting state of the surface from Wenzel state to Cassie–Baxter state. However, when the water contact angle on the smooth surface is smaller than 85.1°, such influence of pillar dimensions on the wetting state was abolished.^84^

Hydrophobicity is one of the most significant properties in material surface science. The hydrophobicity of hydrogels can affect their performance in different applications critically. For example, the delivery of hydrophobic drugs by hydrogels has been limited, as hydrophobic drugs are generally less compatible with hydrogels due to the hydrophilic matrix of hydrogel polymers.^85,86^ By altering the hydrophobicity, hydrogels could be adapted to be able to expand their application in hydrophobic drug delivery as well. Also, it has been demonstrated that hydrogel hydrophobicity can modulate cell behaviors, such as cell adhesion and migration.^87,88^ Inspired by the topographical effect on hydrophobicity and wettability of various materials, such as silicon^83^ and aluminum,^89^ Cutiongco *et al.* measured the water contact angle of cast PVA hydrogel with different topographies. Among several patterns including pillars, concave lenses, and gratings, 2 *μ*m gratings showed significantly higher contact angle compared to flat hydrogel samples.^23^ Similarly, cast pHEMA hydrogel with lotus leaf topography has been measured to have much higher water contact angles compared with flat hydrogel samples.^81^ Another test was also performed on the pHEMA hydrogel. In the test, the water droplet was replaced by a Ga/In/Sn liquid alloy, because the water was immediately incorporated by the prepared hydrogel network. However, it still showed some interesting phenomenon related to the effect of surface microstructure on the liquid state. The pHEMA hydrogel was structured to have 165 × 170 *μ*m^2^ rectangular pillars with 1500 *μ*m height and 700 *μ*m center-to-center distance. Compared to the smooth pHEMA surface, the liquid contact angle on the patterned pHEMA surface was significantly higher.^90^ The above studies show that the surface topography has an effect on hydrogel material hydrophobicity, which supports further research on commercial hydrogel products.

### B. Surface topography alters protein adsorption on hydrogels

As a critical component in human body fluids, proteins can adsorb onto the surface of the material within seconds, once being exposed to a biomaterial.^91^ Such adsorption is essential in inducing cell responses;^92,93^ on the other hand, the adsorption can lead to unexpected pathological phenomenon. For example, the adsorption of blood proteins on blood-contacting biomaterials can trigger the activation of coagulation and complement pathways, followed by blood cell activation, which will lead to thrombus formation on the surfaces.^10^ Also, in the area of contact lens research, adsorption of tear film substances onto the lens material, including proteins and lipids, can lead to wearer discomfort or even severe eye symptoms.^94^ Developing biomaterials with the ability to prevent unspecific protein adsorption will be significant for anti-fouling surfaces, and other applications with defined chemistry or with specific and desirable bioactivities.

Recent studies have shown that adding topography onto hydrogel surface can alter protein adsorption. PEG is reported to be protein- and cell-repellent. Schulte *et al.* formed hydrogel with 6-arm star-shaped poly(ethylene glycol) (star-PEG) macromonomers by UV lithography. Both flat star-PEG hydrogel and patterned star-PEG hydrogel samples were washed in sterile water and PBS to remove toxic residuals before fibroblast cell culture. Two patterns were selected, pillars with 3 *μ*m diameter, 3 *μ*m height, and 6 *μ*m center-to-center distance and lines with 5 *μ*m depth and different spacing distances from 5 to 50 *μ*m. No cell spreading was observed on the flat hydrogel surfaces as expected, while on the patterned surfaces, cells spread on pillar tops and wrapped around the structures. One possible reason why cell adhesion was successful in patterned PEG was that the amount and type of proteins adsorbed on the structured areas were different from that on flat surfaces. To further support this hypothesis, they continued experiments on the adsorption of proteins onto patterned hydrogel surfaces, including bovine serum albumin (BSA), bovine fibronectin (FN), and bovine vitronectin (VN). Both bovine FN and bovine VN showed a preference to adhere on the groove walls on surfaces with line patterns.^95,96^ Similarly, Cutiongco *et al.* reported that the human umbilical vein endothelial cells (HUVEC) had significantly higher adhesion on cast cyclic RGD peptide (cRGD) modified PVA hydrogel films with 2 *μ*m gratings than the unpatterned control. The result again showed the possible effect of surface topography on protein adsorption.^97^

## IV. INFLUENCE OF SURFACE TOPOGRAPHY OR THE PATTERNING PROCESS ON HYDROGEL MECHANICAL PROPERTIES

Mechanical properties of a hydrogel, such as stiffness, strength, and elasticity, can be tuned by adjusting polymer concentration, precursor molecular weight, cross-linking methods, and cross-linking density to meet the requirements in various application fields. The modulus of hydrogels is usually within the range of 10^0^ to 10^4^ kPa.^98^ Generally, surface topography will not change the material stiffness. The relative modulus is mainly determined by the modulus of the bulk material unless the features are high-aspect-ratio pillars.^99–101^ Surface patterning of hydrogels has been shown to alter the surface properties of hydrogel without compromising the mechanical properties. For example, flat and patterned star-shaped poly(ethylene oxide-stat-propylene oxide) hydrogel [Acr-sP(EO-stat-PO) hydrogel] samples (range of modulus 100 kPa–1 MPa) were prepared by casting from micropatterned and blank silicon masters, respectively. Patterns were 10 *μ*m height gratings with different widths ranging from 5 to 50 *μ*m. No significant difference was observed between the stiffness of patterned and blank samples, and the only factor that can alter the hydrogel stiffness was the cross-linking density, which could be controlled by adding different amounts of cross-linking agent and photo initiator.^102^

However, the mechanical properties of hydrogels can also be manipulated via the patterning processes as part of the design. A digital plasmonic patterning method which was developed to pattern PEG hydrogels has been shown to directly vary the hydrogel stiffness from 17 to 350 kPa by controlling the laser intensity and the writing speed.^103^ Similarly, poly(ethylene glycol) diacrylate (PEGDA) hydrogel (100 kPa) was reported to become stiffer after patterning with photolithographic patterning technique. The pattern stripes were fabricated in a way that low molecular weight PEGDA molecules diffused and crosslinked into the high molecular weight PEGDA hydrogel network under the predesigned photomask. As a result, the stiffness of the patterned area was higher than the base hydrogel, and the whole patterned PEGDA hydrogel samples also showed higher stiffness along the pattern stripe orientation.^104^ Electrospinning is another way to produce a hydrogel matrix with anisotropic mechanical property.^105,106^ For example, the anisotropic collagen hydrogel (456 kPa for aligned scaffolds and 349 kPa for random scaffolds) can be fabricated from the hydrogel's anisotropic contraction by lyophilizing the collagen solution repeatedly.^107^ These designs make it possible to fabricate hydrogels with different mechanical properties in different local regions, and the cell response can be further studied on such hydrogel because stiffness can direct the cell behaviors. Classical mechanical measurement methods, such as static tensile/compression tests, are generally more suitable to characterize the hydrogel mechanical properties in macroscopic scale,^108^ while an atomic force microscopy (AFM)-based method called force spectroscopy mapping (FSM) can provide more microscopic information on the anisotropy of hydrogels. Two hydrogels with similar bulk roughness and stiffness have been demonstrated to have a significant difference in their nanomechanical properties.^109^ Therefore, it is essential to develop a better understanding of how hydrogel mechanical property can be influenced by surface topography.

In addition to the bulk material mechanical properties, studies in the literature have also demonstrated the aspect ratio of topographical features could change the effective substrate stiffness or the relative mechanical properties that would be sensed by cells interacting with the materials.^110,111^ For example, the aspect ratio of pillars can affect the effective stiffness of the microarray of the pillar. The bending force was reported as F = (3EI/L^3^)δ, where *F*, *E*, *I*, *L*, and *δ* are the bending force, Young's modulus, moment of inertia, length, and resulting deflection of the post, respectively in Tan *et al.*,^112^ or as F = (3/4πE(r4/L3)), where *r* is the radius of the pillar, *L* its height, *E* Young's modulus, and Δ*x* is the deflection of the post, respectively, in du Roure *et al.*^113^ The mathematical relationship between the Young modulus of the materials and the bending or collapsing force of the patterned features has been developed.^68,100^ As discussed in Sec. II B, the aspect ratio and mechanical properties would also affect the maintenance of structure fidelity. The reader is referred to a study by Chandra and Yang for extended reading on the stability of high-aspect-ratio micropillar array.^68^ In Secs. V and VI of this review, we will focus on discussing the topographical features with aspect ratio of height to width around or less than 2 and their influences on microbial adhesion and mammalian cell interaction.

## V. SURFACE TOPOGRAPHY AFFECTS MICROBIAL ADHESION TO HYDROGELS

Microbial adhesion or biofilm formation on medical devices could lead to serious health problems. Patients can suffer from infections or even death with pathogenic bacteria adhesion on medical devices, such as implants and catheters. In recent years, several methods have been developed to reduce or prevent microbial adhesion of biomaterials, including adding antimicrobial reagents or toxic biocides into coatings and substrates.^114,115^ However, such toxic reagents added into the biomaterials could possibly harm human cells or tissues, especially in close proximity or with close contact. The effectiveness of the biocides could also be unstable for biocides with a short half-life.^116,117^ In order to provide a safer microenvironment for medical use, numerous efforts have been made to develop a more efficient and user-friendly technique that can reduce microbial adhesion. Surface roughness and surface topography are factors that are newly discovered to be able to significantly affect the interactions between bacteria and material surfaces. Both of them have been applied on biomaterials to control microbial adhesion in biomedical applications. The effects and mechanism of each type of surface modification are different, and they are further discussed below.

### A. Surface roughness effect

Surface roughness mainly shows heights and depths of surface irregularities, which can be measured via two parameters R_a_ and R_rms_, respectively. Yong *et al.* tested the adhesion of *Staphylococcus aureus* and *Pseudomonas aeruginosa* onto the Etafilcon A hydrogels with different surface roughness values. A significant positive correlation existed between the hydrogel roughness and colony forming units (CFUs) of the two bacteria.^118^ Similarly, *Staphylococcus epidermidis* adhesion onto five kinds of hydrogels (Omafilcon A, Ocufilcon B, Nelfilcon A, Senofilcon A, and Comfilcon A) with varied R_a_ and R_rms_ values measured by AFM was studied.^119^ In the result, hydrogels with lower R_a_ values were observed to have lower CFUs, and the authors suggested that it is probably because the colonization of microorganism could be affected by the surface roughness.^119^

**FIG. 4.**
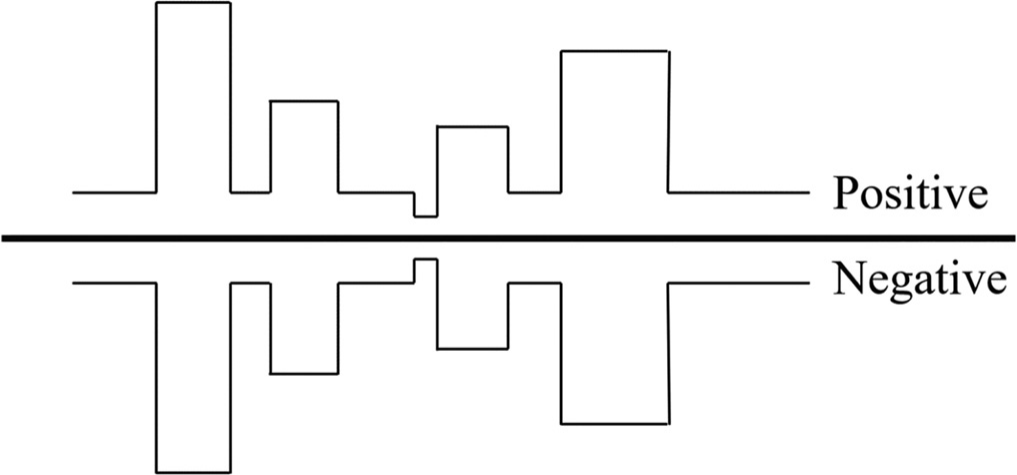
Two opposite surfaces with similar R_a_ values.

**FIG. 5.**
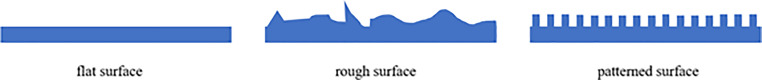
Comparison between a flat surface, a surface with random roughness, and a surface with specific patterns.

However, the two parameters R_a_ and R_rms_ are not sufficient to describe and characterize the surface properties. Only the information about the variation of asperities heights can be given by the surface roughness values. For example, although the shapes, slopes, or sizes of irregularities can be different on two surfaces, the calculated values of R_a_ can still be very similar to each other when values of peaks and valleys are canceled out (Fig. 4).^119,120^ Therefore, these two surfaces with similar roughness value could perform differently in different specific applications. The effect of material surface roughness on bacterial adhesion has been controversial. Some researchers argued that rougher surfaces lead to higher adhesion forces of bacteria, while others argued that the surface roughness had nothing to do with the bacteria adhesion or even prevented the adhesion.^118,119,121–123^ Such a debate also reflects the controversy of the actual effects of surface random roughness. Due to this problem, precisely designed topographies, in which researchers can engineer the dimension, shape, and geometry of the topography systematically, can be more useful and promising in studying how surface patterning affects the interactions between bacteria and biomaterial surfaces (Fig. 5).

### B. Surface topography effect

Bacterial motility on the surface can be led by the interaction between the topography and bacteria appendages, such as flagella and pili. According to the shape and size of the topography, different bacteria also show distinct motion preferences and responses to the surface, such as near-surface swimming and surface-anchored spinning.^124^ Surface topographies can achieve antibacterial functions by providing anti-adhesion surfaces or bactericidal surfaces. Anti-adhesion surfaces aim to prevent bacterial cells from attaching to a surface via unfavorable surface topography. It has been discovered that topographies with smaller sizes work more efficiently to decrease bacterial adhesion than large structures. Bactericidal surfaces refer to surfaces with specific structures, such as closely spaced nanoscale pillars that can directly pierce through the bacteria cell membrane and kill the bacteria within several minutes.^116,125^

In nature, many animals or plants have evolved surfaces with specific topographies that can either support self-cleaning or protect themselves from bacteria. Such inherent functional surfaces provided inspiration in applying these bio-inspired micro/nanostructures into other synthetic materials to give them antibacterial properties.^126–129^ Nanopillars on the wings of cicada (*Psaltoda claripennis*) with a height of 200 nm and center-to-center distance of 170 nm have been demonstrated to be able to puncture the membranes of *P. aeruginosa* and kill them within 3 min.^130^ The inner and outer membranes of *Escherichia coli* were damaged and separated from each other on dragonfly (*Orthetrum villosovittatum*) wings due to the existence of nanopillars with heights in the range of 189 to 311 nm and diameters in the range of 37 to 57 nm.^131,132^ Black silicon surfaces with similar biomimicking high-aspect-ratio nanofeatures could kill *S. aureus* and *P. aeruginosa* bacteria effectively at an estimated killing rate of 450 000 cells min^−1^cm^−2^.^116,133^ The adhesion of *E. coli* and *S. aureus* on micropatterned PDMS were also observed to be reduced when the bacteria size is larger than that of the pattern groove.^125,134^ Microbial adhesion on more rigid materials with surface topography, such as implant topography, has also been extensively studied. However, as the current paper focuses on topography on hydrogel, readers can refer to excellent review papers for further extended reading.^135–137^

**FIG. 6.**
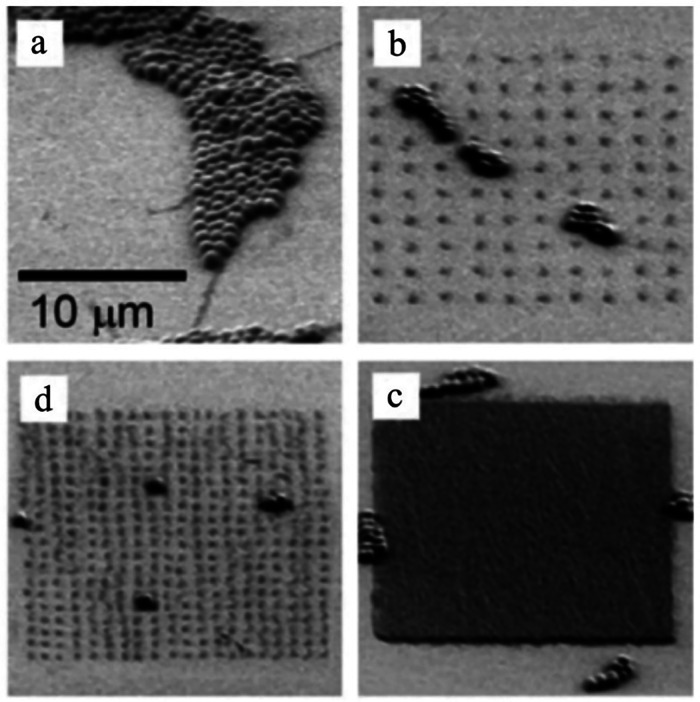
*S. epidermidis* adhesion on PEG hydrogel with different patterns: (a) blank control, (b) 2 *μ*m apart, (c) 1 *μ*m apart, and (d) 0.2 *μ*m apart. Reprinted with permission from Krsko *et al.*, Acta Biomater. **5**(2), 589–596 (2009). Copyright (2009) Elsevier.^142^

As a popular biomaterial, hydrogels with organized surface textures have also been fabricated to study their antimicrobial performance. However, most studies are designed to target bacterial adhesion on hydrogels, while adhesion of other microbes, such as fungi or virus, is much less taken into account. *Pseudomonas aeruginosa* was cultured on both cast flat and surface patterned chitosan hydrogel films for 18 h, and CFUs were then counted on agar plates to see if the surface topography could inhibit the bacteria growth.^138^ Compared to the flat hydrogel films, *P. aeruginosa* cultured on nanopillars with 120 nm diameter and 230 nm height showed 31% lower CFUs. Nanopillars with 190 nm diameter and 400 nm height exhibited even better antibacterial property with 52% lower CFUs compared to flat chitosan films. The adhesion of *E. coli* onto the patterned PEG hydrogel was examined in another study.^139^ In the research by Koh *et al.*, PEG hydrogel with 30 × 30 *μ*m^2^ square microwells fabricated by UV lithography was attached covalently to the silicon substrate surface via a 3-(trichlorosilyl) propyl methacrylate (TPM) monolayer. After incubating the samples with microstructured PEG hydrogel in suspended *E. coli* solution for 6 h, the *E. coli* bacteria were observed to be confined within the three-dimensional trenches of the hydrogel, showing the active resistance of microstructured PEG hydrogel to the *E.coli* adhesion. Similarly, another group also incorporated patterned PEG hydrogel coating onto a silanized glass substrate by e-beam lithography method to study the bacteria adhesion compared with common biomaterials, including silicone rubber, poly(methyl methacrylate) (PMMA), and tissue culture polystyrene (TCPS).^140^ The diameter of the hydrogel pattern was designed to be 2.5, 5, and 10 *μ*m with 5 or 10 *μ*m interpatch spacing distance. *Staphylococcus aureus* was first allowed to adhere onto the samples for 30 min, and the lowest bacteria adhesion was observed on patterned PEG hydrogel coatings. Then, murine macrophages were added to see how different surfaces would affect the phagocytosis of the bacteria. Interestingly, the unpatterned PEG hydrogel coated surface exhibited the lowest phagocytosis rate, but this rate was significantly increased on hydrogel patterned surfaces, depending on the patch diameter and the interpatch spacing. The underlying detailed mechanism was still not clear due to lack of research. However, these studies provide the directions for further research on the relationship between bacteria, macrophages, and patterned surfaces. To prevent bacterial contamination more effectively, Papi *et al.* have combined graphene oxide (GO) hydrogels with *Cancer pagurus* (crab) carapace surface patterns by laser printing, as GO can cause membrane disruption to kill microorganisms and *C. pagurus* carapace is a natural antibacterial surface.^141^ The result again illustrated that the patterns on GO hydrogel surfaces reduced the colony area by around 70% for *S. aureus*, 65% for *E. coli*, and 45% for *C. albicans*. Also, a surface-patterned PEG hydrogel crosslinked on the silanized glass substrate by e-beam lithography has been demonstrated to effectively control the adhesion of *S. epidermidis* and to prevent the development of large bacteria colonies (Fig. 6).^142^

## VI. SURFACE TOPOGRAPHY AFFECT CELL RESPONSES ON HYDROGEL

Cells are surrounded by a complex microenvironment with geometrically defined structures *in vivo*. The extracellular environment provided three-dimensional (3D) physical cues in micrometer and sub-micrometer scale, which plays an essential role in diverse cell processes. To mimic natural extracellular environment, micro- and nanotopographies have been fabricated on substrates and implants to modulate cell processes *in vitro* and regulate cell behaviors *in vivo.* A number of review papers have summarized the cell responses to different topographies.^143–146^

Hydrogels are attractive candidates for cellular studies and tissue engineering application. Due to their high water content, tunable physical and biochemical properties and compatibility with various types of cells, hydrogels can be engineered to resemble native extracellular matrix and generate artificial organs. Various types of hydrogel with rigidity that matches the rigidity of body tissues have also been developed as platform to study mechanobiology. Different cell behaviors, such as desirable cell adhesion, controlled cell migration, increased or decreased cell proliferation, and guided stem cell differentiation, may be required depending on the application fields. These responses can be regulated by altering the biophysical and biochemical properties of a hydrogel. Inspired by the findings of the role of topographies in cell reposes, various dimensions of topographies have been incorporated to hydrogels to mimic native 3D extracellular environment. Examples of topographies on hydrogels for different application including pHEMA hydrogel,^147^ PVA hydrogel,^148^ collagen/gelatin hydrogel,^149^ PEG hydrogel^150^ and polyacrylamide (PAM) hydrogel^151–153^ have been summarized in Table II. In this section, we will discuss how topographies are used to guide cell behaviors, including adhesion and morphology, migration, proliferation and differentiation on hydrogel for different application purposes.

**TABLE II.** Representative studies of cell response to hydrogels with topographical cues.

Hydrogel	Topography		Cell type	Objectives	Key applications	References
	Type	Dimensions				
Poly(hydroxyethylmethacrylate) (pHEMA) (range of modulus: 0.5–1.5 MPa)	Ripples pattern	250–500 nm in height with a width of 3–10 *μ*m	Human corneal epithelial cell (hCEC)	• Increase cell attachment	• Significantly increased hCEC attachment and growth	154
	Lotus leaf	⋯	Human corneal epithelial cell (hCEC)	• Increase cell attachment	• Stronger cell adhesion on patterned hydrogel	81
	Microplate-like feature	2 *μ*m thick, 20 *μ*m height, 10–50 *μ*m wide and the intercolumn spacing was 5 *μ*m	Human mesenchymal stem cells (HMSCs)	• Guide cell orientation and shape	• Cells elongated and aligned parallel to the plates	147
					• Elongation was more pronounced on the patterns with narrower interplate spacing	
Poly(vinyl alcohol) (PVA)-based hydrogel (range of modulus: 0.25–3.7 MPa)	Gratings; pillars; lenses	250 nm, 2 *μ*m, and 10 *μ*m gratings; 2 *μ*m and 10 *μ*m diameter pillars; 1.8 *μ*m, 2 *μ*m, and 10 *μ*m convex lens; 1.8 *μ*m concave lens	Vascular endothelial cells	• Increase cell adhesion	• Cells had significantly better adhesion on 2 *μ*m gratings, 1.8 *μ*m convex and concave lenses	23
					• PVA small diameter vascular grafts with 2 *μ*m grating luminal patterning remained patent, and had good *in vivo* endothelialization	
	Gratings; lenses	2 × 2 × 2 *μ*m^3^ gratings; 1.8 *μ*m concave lenses	Vascular endothelial cells	• Induce aligned morphology	• Cells were elongated on gratings but remain rounded on lens structures	148 and 155
	Squares	100–500 *μ*m^2^ with height of 5, 10 *μ*m, 100 and 200 *μ*m	Dermal fibroblasts and epidermal keratinocytes	• Guide cell migration for wound healing	• Cells migrated from pits to summit	230
Collagen/gelatin (Range of modulus: 5.8–233.3 kPa)	Grooves; steps	6–30 *μ*m in width	Human dermal fibroblasts (HDFs);	• Align cells	• Cells aligned in the direction of grooves with depths of >2 *μ*m.	163
					• Cell aligned poorly on grooves less than	
		0.2–24 *μ*m in depth	Human umbilical artery smooth muscle cells (HUASMCs)			
				• Guide cell migration	• 1 *μ*m in depth	
	Undulation	150–450 *μ*m	Neonatal human fibroblasts (NHFs)	• Induce alignment	• Cells grew in the shape of undulation	30
					• Cells aligned preferentially align to the curvature of undulation	
	Pillars	250 nm and 1 *μ*m pillars with 1 and 6 *μ*m spacing	Human corneal endothelial cells	• Improve cell density	• Cells have a higher density on 1 *μ*m pillars with 6 *μ*m spacing	56
					• Cells had higher Zona Occludens 1 (ZO1) expression on 1 *μ*m pillars	
	Grooves	400 nm nanoridges with 800 nm microgrooves	Human adipose‐derived mesenchymal stem cells	• Induce cell alignment	• Cells aligned with nanotopography	149
	Grooves	5–9 *μ*m	NIH3T3	• Induce cell alignment	• Cell alignment on dynamically imprinted grooves was notably delayed than pre-performed grooves	45
Poly(ethylene glycol) (PEG)-based hydrogel (range of modulus: 60–350 kPa)	Lamellar	⋯	Human palatal mesenchymal cell	• Improve attachment	• Enhanced cell attachment	150
	Wells	500 nm × 4 *μ*m microwell with depth of 400 nm	Preadipocytes	• Induce cell morphology change	• Cells grew into microwells and exhibited more confined morphology	167
				• Direct cell differentiation	• Cells underwent differentiation	
	Pillars; grooves	3 *μ*m pillars with 3 *μ*m height; microgrooves with height of 5 *μ*m and width of 5–20 *μ*m	Fibroblast	• Improve adhesion	• Topography increased cell adhesion	95
					• Stable cell-surface contact formed on grooves with dimension in the cell size or smaller (<10 *μ*m)	
				• Regulate morphology		
					• Cells spread on top of pillars and wrapped around the pillars	
	Lines	Width of 5–50 *μ*m, space of 10 and 50 *μ*m and depth of 5, 10 and 15 *μ*m	Fibroblasts	• Guide cell migration	• Cell migration tracks were random inside wide channels, but parallel on narrow ones	180
	Grooves	100 *μ*m	Human epithelial cells	• Improve migration	• Cells had increased motility on patterned structures	182
					• Relatively upright walls were necessary	
	Grooves	400–4000 nm pitch, 300 nm height	Corneal epithelial cells	• Improve migration	• Cells explored a larger space, migrating on average over 100 *μ*m migrated parallel to the ridge and groove topographies	177
	Wells	Diameter of 40 to 150 *μ*m and height of 20–35 *μ*m	Embryonic stem cells	• Induce cell differentiation	• The microwells can initiate the embryoid body formation	218
Polyacrylamide (PAM)-based hydrogel (range of modulus: 1–145 kPa)	Square posts; hexagonal posts	Varied post size from 1–20 *μ*m with varied gap sizes	Mesenchymal stem cells	• Guide adhesion	• Cells located in the gap when gaps were larger than 15 *μ*m, while located on top of posts when gaps were smaller than 5 *μ*m	168
				• Regulate cell morphology		
					• Cells elongated along narrow gaps	
	Grooves	50 *μ*m width 35 *μ*m depth	Cardiac fibroblasts	• Induce cell alignment	• Cells arranged along the ridges, but soft substrate induced minimal alignment	152
	Grooves	2 × 2 × 2 *μ*m^3^ and 4 × 4 × 4 *μ*m^3^	Fibroblasts	• Induce alignment	• Cells form protrusions in the grating grooves; focal adhesions were aligned to the grating direction	221
	Grooves; hexagonal and square pillars	Microgrooves with 5 *μ*m depth, 2 *μ*m ridge width, and 15 *μ*m ditch width, hexagonal pillars with 5 *μ*m ridge width and 15 *μ*m side-length, and square pillar with 10 *μ*m side-length and 10 *μ*m interpillar gap	Human embryonic stem cells	• Regulate morphology	• Cells formed flattened colony on a groove or pillar substrate but spheroid colony on a hexagonal substrate.	219
	Square pillars and grooves	5, 10, and 15 *μ*m pillars and grooves	Rat bone marrow mesenchymal stem cells (rBMSCs)	• Regulate morphology	• Cells on pillar substrate formed a large spherical shape	166
	Groove	Rectangular grooves with 10, 15, and 25 *μ*m in width	HMSCs and mouse embryonic stem cells (mESCs)	• Induce neuronal differentiation	• Soft 5 kPa gels containing 10/15 *μ*m grooves induced strongest neuronal marker expression of hMSCs	204
					• mESCs are unable to sense topographical features when cultured directly on grooved gels	
					• Soft substrates are essential for inducing topography-mediated neuronal differentiation in mESCs	
	Grating	Widths between 20 and 200 *μ*m	Epithelial cluster	• Induce epithelial-to-mesenchymal transition	• Grating structures confined epithelial cluster, which induced epithelial-to-mesenchymal transition through cytoskeletal polarization	153
	Grating	Nanogratings with width of 300 nm, height of 600 nm and space of 600 nm	Macrophage	• Reduce inflammatory response	• Hydrogels with gratings of 600 nm n space showed lower number of neighboring macrophages and lowest thickness of encapsulation	151

### A. Improve cell adhesion and regulate morphology

Cell adhesion is essential in cell communication and signaling. However, adhesion of cells on hydrogels that lack of cell binding anchorage or do not support ECM adsorption is challenging. Topographical modification is one of the commonly used modifications on hydrogel surfaces that have been used to enhance cell adhesion on non-adhesive hydrogels.

**FIG. 7.**
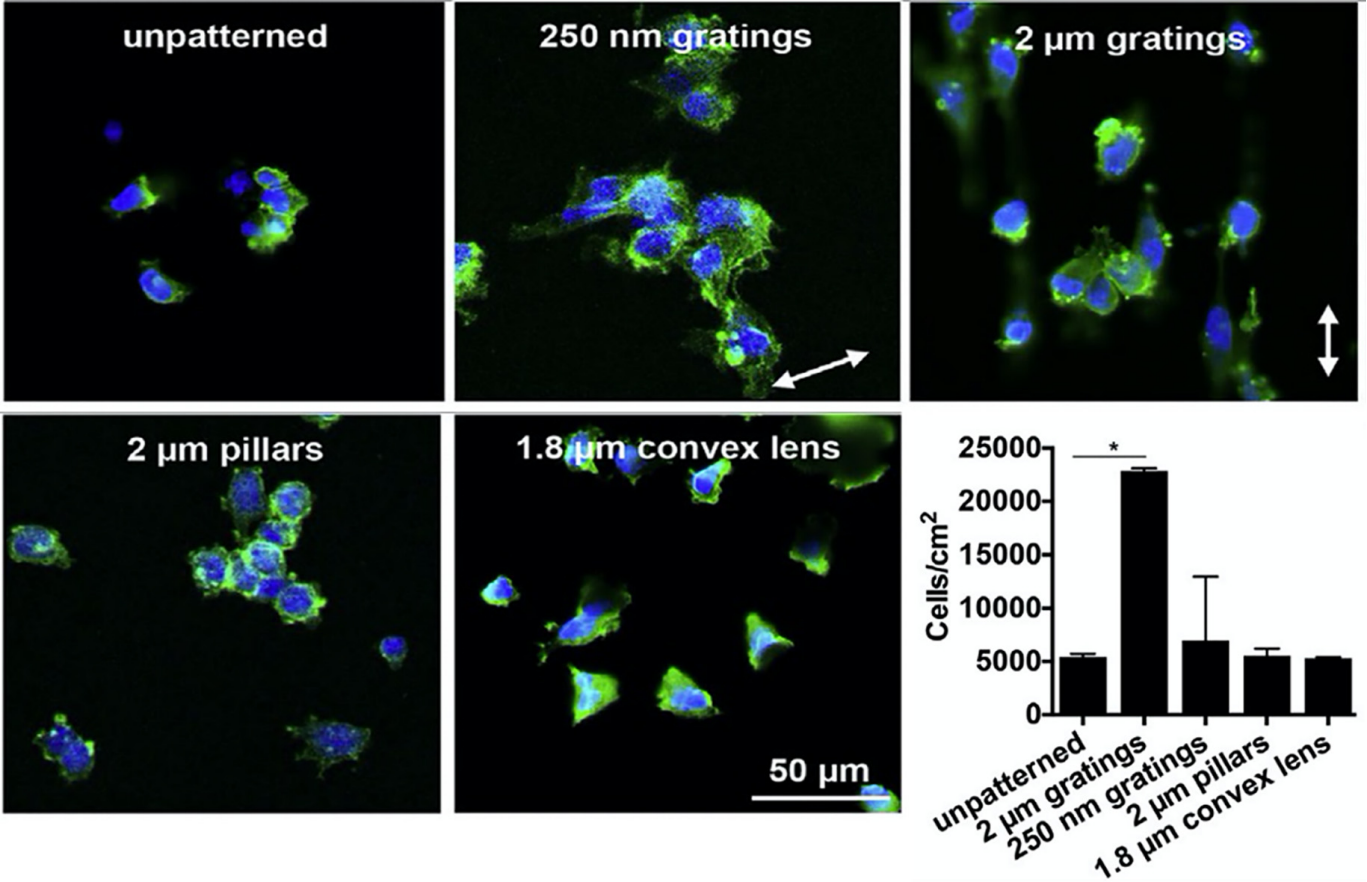
Human umbilical vein endothelial cell adhesion on polyvinyl alcohol (PVA) hydrogels with different topographies. Reprinted with permission from Cutiongco *et al.*, Biomaterials **84**, 184–195. Copyright 2016 Elsevier.^23^

Poly(2-hydroxyethyl methacrylate) (pHEMA) is a commonly used hydrogel for contact lens. Nanosized rippled patterned^154^ and Lotus leaf topographies^81^ have been introduced to pHEMA by laser treatment and casting methods, respectively, to increase human corneal epithelial cell attachment and growth. Poly(vinyl alcohol) (PVA) is a biocompatible material and has shown potential for small diameter vascular graft; however, the lack of cell adhesion sites limits its application. Our group has developed patterned PVA hydrogels with different dimensions of isotropic and anisotropic topographies by casting and nanoimprinting methods.^23,155^ We found that vascular endothelial cells had substantially enhanced attachment on 2 *μ*m gratings both *in vitro* and *in vivo*, while had minimal attachment on unpatterned PVA, as shown in Fig. 7. Cells can sense topographies from nanometer to micrometer scale. The promotion effects of topographies on cell adhesion are dependent on topography dimensions. Hepatocytes attachment on heparin hydrogels with gratings of different pitch sizes, fabricated by UV lithography, were compared, and gratings with height of 300 nm and pitch of 400 nm supported markedly better attachment.^156^ Similarly, fibroblasts exhibited good adhesion on polyethylene glycol hydrogels with 3 *μ*m pillars and grooves prepared by casting method,^95^ and grooves with 10 *μ*m in width, which is in the range of the cells' own size, induced significantly better cell adhesion and spreading.^102^

In addition to cell adhesion, substrate topographies can also induce cell morphology change, cell alignment and cytoskeletal re-arrangement. Hydrogels with aligned microfibers and nanofibers were well documented to induce cell alignment along the fibers.^157–159^ Alginate hydrogels made by wet spinning were exposed to shear force to reshape the hydrogel fiber into aligned sub-micrometer topography.^95^ Cells were shown to orient along with fiber axis and formed cell-matrix dual alignment. 3D laminin-rich matrices with alignment fibers were also shown to induce cell alignment.^157^ The aligned cells showed extended protrusions parallel or perpendicular to aligned fibers, and the focal adhesion mainly diffused in the cytoplasm, with few puncta localized at the protrusions.^157^ Similarly to aligned fiber, hydrogels with micro- and nanogroove structures were also effective in inducing cell alignment parallel to the grooves.^45,147,149,160–162^ The dimension of grooves was shown to affect cell alignment differently. Robert *et al.* showed that hydrogels with 1.9 *μ*m of grooves by casting method were effective in inducing cell alignment, but cells aligned poorly on grooves with depth less than 1 *μ*m.^163^ Adipose-derived stem cells were also shown to exhibit alignment when the topography width is larger than 0.60 *μ*m and height larger than 170 ± 100 nm on elastin-based hydrogel.^164^ Fibroblasts were cultured on PAM hydrogels with 5 *μ*m wide and 1 *μ*m high lines with stiffness of 13, 37, and 145 kPa. Cell elongation was induced by topography on all substrates. Topography-induced elongation was more obvious on stiff substrates. Primary intestinal epithelial cells were cultured on patterned substrate with stiffness (13 kPa) comparable to basement membrane and stiffer substrate (145 kPa). Cells spread more on harder hydrogels and the epithelial clusters expanded a twice larger area on stiff substrate than soft substrates.^165^ Cells on microwell and micropillar structures exhibited distinct morphology compared to on grating structures. Rat bone marrow mesenchymal stem cells were shown to form a large spherical shape in a pillar substrate but not in a grooved substrate fabricated by lithography.^166^ Preadipocytes on poly(ethylene glycol) (PEG) hydrogel with imprinted nanowell structures exhibited spherical but more confined shape compared to unpatterned surfaces.^167^ Al‐Haque *et al.* investigated the responses of cardiac fibroblasts to topographies on both soft and stiff polyacrylamide (PAM) hydrogels. Cells on substrates of intermediate stiffness (18 and 50 kPa) had most significant topography-induced cell elongation. Cells on soft substrate (1 kPa) were also able to elongate along the topography, while cells on stiff substrates (143 kPa) did not exhibit appreciable topography-induced elongation.^152^

Hydrogels have also attracted broad interest for use as *in vitro* cell culture scaffolds. Recent studies have introduced microscale topographies on hydrogel cell culture scaffolds to control cell location and configuration. Mesenchymal stem cells cultured on PAM hydrogels with an array of microposts with varied shape, width, and spacing prepared by casting method were studied.^168^ Cell bodies tended to locate in 15 *μ*m and wider gaps while located on top of posts that were 5 *μ*m and smaller. Cardiomyocytes were found to be confined within 50 *μ*m microgrooves on gelatin methacryloyl (GelMA) hydrogels fabricated by photo nanoimprint and formed uniform and highly aligned cardiac tissues.^169^ This modulation behavior of topographies on hydrogels made it suitable for single cell arraying and controlled cell culture. Pasturel *et al.* have designed a light-based toolbox to photoprint hydrogel topographies, which work as templates to direct cells to grow and self-organize into standardized structures.^170^ Gelatin-based hydrogels with microsized undulation topography by casting method was shown to be suitable for cells to grow in the shape pf undulation and formed multiple monolayers to resemble skin.^30^ Non-adhesive hydrogels with programmable geometries have also shown the capability to control self-organization of cellular aggregates.^171^ In addition, chitosan hydrogels with microwells prepared with molding processes facilitated the co-culture of hepatocyte spheroids and fibroblast monolayers, enabling the study of heterotypic cell–cell interaction.^172^

### B. Direct cell migration

Substrate topographies have been documented to provide contact guidance, accelerating cell migration, which has been mainly observed on surface with groove structure or on aligned fibers.^143,173–175^ Mechanism underlying this phenomenon has been proposed to be topography-induced geometry constraint of cell adhesion sites, which results in cell alignment, polarization and directional migration. Review papers from Petrie *et al.*^173^ and Anselme *et al.*^176^ have summarized studies about the effects of topographies on cell migration. Inspired by those findings, researchers have also fabricated aligned fibers and grooved structures on hydrogels to increase the directional cell migration both *in vitro* and *in vivo.*^177–179^

PEG-based hydrogel with microgrooves prepared by casting increased the rate of corneal epithelial cell migration *in vitro.*^177,178^ Corneal epithelial cells on microgrooved substrates were found to explore larger space and migrated on an average of 100 *μ*m parallel to the ridge and groove topographies.^177^ They also exhibited 50% higher wound healing rate compared to unpatterned surfaces.^178^ Electrospun fibrin hydrogel with 3D hierarchically aligned fibers were implanted in a rat dorsal hemisected spinal cord injury model to study its function in spinal cord injury recovery.^179^ Accelerated directional host cells invasion along the fibers *in vivo* was observed in the first week after surgery, and the locomotor performance of the aligned fibrin group recovered much faster than random fibrin hydrogel. The efficiency of grooved structure in promoting cell migration was shown to be dependent on groove width and the slope of groove walls. Vicente *et al.* found the orientation of migration tracks with pattern appeared to increase with the decreasing of linewidth.^180^ Cells were shown to migrate randomly inside wide channels that were larger than cell size, while on narrow channels, cell migrated parallel to the pattern direction. Fibroblasts appeared to adhere, align, and elongate more on denser patterns on polyurethane-amide (PUA) hydrogels with variable groove width of 1–9 *μ*m prepared by UV-assisted capillary molding.^181^ The migration speed of cells was affected by pattern density with the fastest speed frequently occurring at intermediate ridge density. Epithelial cells were shown to increase their motility by threefold on the microgrooved PEG hydrogel prepared via casting than non-patterned hydrogels.^182^ By varying the slope of the microgroove walls, the authors found that relatively upright walls are necessary for increased cell migration.^182^

### C. Alter cell proliferation

Cell proliferation is regulated by the extent and strength of cell adhesion and was reported to be positively correlated with cell flattening.^183^ Substrate topographies play a role in cell proliferation through by affecting cell spreading on the substrates. However, different cell types exhibited distinct proliferation responses to topographies. Microsized circular topographies on epoxy resin and poly(dimethylsiloxane) (PDMS) were shown to be promising in controlling epithelial cell proliferation.^184,185^ Corneal endothelial cells were shown to proliferate significantly faster on micropillars on PDMS^186,187^ and tissue cultured polystyrene (TCPS).^186,188^ The proliferation rate of vascular endothelial cells were not significantly affected by topographies on PDMS,^189,190^ while that of smooth muscle cells was shown to be reduced on nanogrooved structures.^175^ A detailed review by Anselme *et al.* listed examples of various cell proliferation responses to substrate topography.^176^

Based on these findings, topographies have been incorporated on hydrogel scaffolds to improve or suppress cell proliferation depending on different application purposes. Silk-graphene hybrid hydrogels with aligned nanofibers were shown to have preferable stiffness for nerve cell study.^191^ Proliferation of multiple nerve cells was shown to be promoted by the aligned fibers on the hydrogels, indicating the potential of this hydrogel for use as platform for nerve regeneration. Electronspun fibrin nanofiber hydrogels with hierarchically aligned fibers were designed to promote peripheral nerve regeneration.^159^ The nanofibers were shown to have the capability to direct Schwann cells migration and proliferation and accelerating axonal regrowth.^159^ On the contrary, microsized gratings seem to hinder the proliferation of smooth muscle cells. Human aortic smooth muscle cells had significantly lower proliferation on microgrooved tetronic-tyramine hydrogels (10, 25, and 80 *μ*m) prepared by casting method than unpatterned hydrogel, independently from groove size.^192^ Human corneal endothelial cells were seeded on GelMA+ hydrogel, which was sequential hybrid crosslinked with physical followed by UV cross-linking to achieve stronger mechanical strength. GelMA+ with 1 *μ*m pillar structures prepared by capillary force lithography had higher cell density compared to the unpatterned control.^56^ To study cell responses to multiple stimuli, patterned PVA hydrogels prepared by casting with different stiffness were used as scaffolds to study human pancreatic cancer cell responses.^193^ Cells exhibited significantly better adhesion and proliferation on nanopillars structures on fibronectin functionalized PVA hydrogels, and the cells appeared to favor nanopatterned surfaces over micropatterned and flat surfaces.^193^ A recent study also studied corneal endothelial cells responses to hexagonal patterns on PAM hydrogels with stiffness comparable to native Descemet's membrane. Cells on small patterns (2000 hexagons/mm^2^) had significantly higher proliferation rate than those on large patterns (400 hexagons/mm^2^).^194^ In addition, topographies have also been incorporated in 3D cell culture scaffolds to maintain desired cell viability, proliferation, and maturation. 3D PAM hydrogel cell scaffolds with hexagonally ordered spherical cavities with diameter of 97 *μ*m were shown to be suitable for *in vitro* 3D cell culture.^195^

### D. Control cell differentiation

Stem cells have emerged as important cell source for regenerative medicine due to their differentiation and self-renewal capability. Stem cells have been demonstrated to respond to biophysical and biochemical cues in their natural niche. Stiffness is considered as a key parameter in the microenvironment that directs cell differentiation, and the underlying mechanisms have been discussed in several reviews.^196,197^ Topography is another key feature that can be harnessed to provide 2D and 3D niche to direct cell fate. The influence of topography features, such as geometry, size and curvature, on stem cell fate has been extensively reviewed.^198–201^

The stiffness of hydrogels can be adjusted by changing parameters, such as polymer concentration and cross-linking density, and thus hydrogels have been used as platforms for studying cell differentiation. Hydrogels with topographies showed potential for culturing stem cells and providing niche for directed stem cell differentiation *in vitro* and *in vivo*. Microgrooved structures have been documented to induce neuron differentiation.^202–204^ Neuron differentiation of human embryonic stem cells was shown to increase as groove pitch decreased, and 2 *μ*m microgrooves can improve neuron growth by 1.7-fold.^203^ Sthanam *et al.* compared neuronal differentiation of human mesenchymal stem cells (hMSCs) and mouse embryonic stem cells (mESCs) on microgrooved PAM hydrogels prepared by casting. hMSCs maximally elongated and expressed neuronal markers on soft 5 kPa gels containing 10/15 *μ*m grooves. However, mESCs were unable to sense the topographies when cultured directly on grooved gels. The authors introduced a priming step where the mESCs were cultured on a soft 1 kPa flat gel for 7 days before replating the cells onto the grooved gels. With the priming step, neuronal differentiation was improved in mESC, and the authors suggested that soft substrates are essential for inducing topography-mediated neuronal differentiation in mESCs.^204^ The observations were in agreement with earlier studies to show that cytoskeletal contractility is essential for topography-sensing and topography-induced neuronal differentiation of human ESCs.^205^ Undifferentiated pluripotent stem cells including hESCs^205^ and mESCs^206^ have lower acto-myosin contractility compared to differentiated cells, while the acto-myosin contractility increased during differentiation process. This explained why a priming step in the study by Sthanam *et al.* could help to rescue or promote the mESC topography sensing and differentiation on the grooved gel. Aligned fibers also enhanced neuronal differentiation. Hierarchically aligned fibrillar fibrin hydrogel prepared by electrospinning was shown to induce cytoskeletons alignment of human umbilical cord mesenchymal stem cells (hUMSCs), upregulate neural lineage specific markers, and encourage rapid neurite outgrowth.^207^ In addition to groove structures, nanopillars were also found to be promising to enhance neural stem cell differentiation and regulate neurite outgrowths.^208^ In addition to neuronal differentiation, micro- and nanogroove structures have been shown to promote osteogenic differentiation,^209,210^ myogenesis and myotube alignment,^211^ and chondrogenic differentiation.^212^

*In vitro* stem cell expansion, especially pluripotent stem cells, is frequently required to scale up cell production while maintaining pluripotency. Conventional stem cell culture requires feeder layers or addition of growth factors.^213,214^ Novel methods have focused on using topographical cues to retain pluripotency of stem cells.^213,215–217^ PEG hydrogels with microwells of 40 to 150 *μ*m in diameter and 20–35 *μ*m in height prepared by capillary force lithography can initiate embryoid bodies. The embryoid bodies generated on the patterned substrates remained viable with controllable size and shape and could be easily harvested.^218^ Lü *et al.* studied the stemness of mESCs on PAM hydrogels with microgrooves and square micropillars prepared by soft contact lithography.^219^ The results showed topography manipulate stemness of mESCs via the formation of different shapes of colony. Groove or pillar substrate induced a relatively flattened colony, while a spheroid colony was preferred on a hexagonal substrate. The role of topography in retaining cell stemness was found to be more effective in retaining cell stemness on stiff, hexagonal, or pillar-shaped substrates.

The mechanisms of regulation behaviors of topographies on cells on hydrogels have been studied. As discussed in Sec. IV, the topography alters protein adsorption on hydrogels. This topography-directed protein adsorption was reported to contribute to the improved cell adhesion. The presence of serum proteins was speculated for improved cell adhesion on patterned PEG hydrogels.^96^ The presence of serum proteins, especially vitronectin, in culture medium was shown to be essential for initial cell attachment and topography is important for establishing durable adhesion and cell spreading. In addition, topography-induced differentiation have also been observed to associate with changes in cell adhesion and morphology, which could be due to geometry-dependent cytoskeletal arrangement,^95^ changes in actomyosin contractility,^205^ and focal adhesion signaling.^220^ Actin filaments preferentially form and elongate along the directions with least resistance, and consequently leads to aligned cell shape. Similar to the cells on other patterned substrates, the role of focal adhesion formation has also been stressed when investigating the mechanism of cell elongation on hydrogels with grating structures. Yip *et al.* reported that fibroblasts formed protrusions in the grating grooves on a polyacrylamide (PAM) hydrogel with 2 *μ*m gratings.^221^ Focal adhesions also aligned parallel to the gratings, which also resulted in aligned actin stress fiber formation in the direction parallel to the grating, leading to polarized traction stresses which drive cell elongation. Smooth muscle cells cultured on microgrooved tetronic-tyramine hydrogels were reported to form localized focal adhesions on the ridges of grooves and less organized focal adhesions in the 2 *μ*m depth of the grooves, which contributed to the alignment of actin networks along the grooves.^192^ Similarly, human mesenchymal stem cells also formed long and aligned focal adhesion on 3D printed microchanneled gelatin hydrogels, while formed small and randomly distributed focal adhesions on unpatterned hydrogels.^222^ In addition, the roles of integrins have been emphasized in topography induced cell responses.^223,224^ Micro- and nanosized topographies have also been elucidated to promote integrin cluster formation between cells and the extracellular matrix.^225,226^ The authors would like to introduce recent studies on the topography-sensing mechanism of mammalian cells. However, the focus of the paper is about patterning of hydrogel, we would refer readers to other excellent recent review papers on mammalian cells.^176,227–229^

## VII. APPLICATION OF SURFACE TOPOGRAPHY IN ACTUAL BIOMEDICAL PRODUCTS

While the influence of surface topography on hydrogel surface properties is still far from being fully understood, this method has already been introduced into certain biomedical products to improve their efficacies.

Wound dressing works to protect damaged skin from dehydration and infection. Traditional wound dressing methods, such as cotton wools, bandages, and gauze dressing, can provide significant support in the initial stage of wound healing. However, the removal of these dressing materials often strips off the newly formed epidermis. They are also unsuitable to be used on effectively debrided wounds due to the nonselective debridement.^231,232^ To overcome the drawbacks of the traditional wound dressing, many new materials have been developed, and hydrogel is one of them. Hydrogels can not only keep the moisture content of the necrotic tissue, but also facilitate autolytic debridement by increasing the production of collagenase.^233^ For example, graphene hydrogel has drawn attention as a promising candidate for wound dressing due to its high water absorption, excellent biocompatibility and pain reduction effect.^114,232^ To further increase the healing efficacy, different surface topographies have been applied. A prototype hydrogel wound dressing was surface patterned by casting from the Si mold with different column structures. All these microfeatures on the hydrogel surface showed the ability to protect the adherent cells from shear damage, among which column structures with 250 and 500 *μ*m width exhibited the best performance that more than 80% of the initial cell population was retained, while on blank hydrogel samples, only 35% of the initial cells survived.^230^ Similarly, an alginate/poly-L-ornithine/gelatin hydrogel with 10 *μ*m gratings surface structure was investigated on its ability to enhance wound healing. The features can not only prompt endothelial cell proliferation but also encourage the secretion of growth factor PDGFB.^234^ However, these studies did not evaluate the antimicrobial performance of these surface textures. Ruiz *et al.* also pointed out that graphene hydrogel does not show antibacterial properties, so contamination with microbes, such as gram-negative *Escherichia coli* (*E.coli*) and gram-positive *S. aureus*, and wound infection can occur.^9,235^ To solve such a problem, silver nanoparticles and iodine were still incorporated into the graphene hydrogel and the prototype hydrogel as the antimicrobial agent to increase antibacterial ability, respectively.^114,230^ As the hydrogel wound dressing can generally provide higher user comfort and reduced pain, they will be more popular if the surface topography modification could maximize the wound healing and minimize the microbial contamination simultaneously.

Another example is contact lens. Nowadays, soft contact lenses are commonly used in vision correction, and colored contact lenses are also used for decorative and cosmetic applications. In addition to wearing comfort, potential risks and health threats of contact lens wear, such as microbial contamination, have also been known but yet to be addressed.^236,237^ The temperature that is close to body temperature and hydrated environment on surface of hydrogel contact lens provides a suitable environment for bacterial adhesion and biofilm formation. The proteins, mucin, and lipids from tear fluid could deposit onto the contact lens surface during wear, supporting the formation of biofilm and making it difficult to eliminate the bacteria.^236,238^ Many studies have shown that both gram-positive *cocci,* such as *Staphylococci cocci,* and gram-negative rods, such as *P. aeruginosa,* which have been isolated from worn contact lenses, have been associated with keratitis.^239^ The microbial keratitis can lead to eye pain, excessive tearing, and even severe vision impairment. As soon as the contact lens meets a fluid, such as tear, both bacteria and organic matter will diffuse toward the surface of the contact lens. The organic matter diffuse faster than bacteria because of their smaller size, thus forming a “conditioning film” for bacteria adhesion. The excreted exo-polymeric substances gradually change the initial reversible bacteria adhesion to irreversible adhesion. The growth of infectious biofilms can also be sustained when the contact lens is in contact with the human cornea for a long time.^119,239^ Again, in addition to adding extra antimicrobial agents, roughening or patterning the hydrogel contact lens surface could be a possible way to prevent the microbial contamination.^119^ Due to the potential commercial value of the application and market competition between companies, most of the studies on contact lens surface patterning are patented. For example, a US patent shows that different regions on the contact lens surface could be patterned with different microstructures, such as microwells and microchannels. The dimensions of the features are all less than 200 nm, and they have been tested to increase the lubricity during eye blinking and demonstrate no influence on the optical clarity.^240^ Another Japanese patent in 2012 also showed how the negative effects on the light transmittance can be reduced by adding nanoscale patterns onto their hydrogel lens surface.^241^ The above published patents show that it is feasible to improve the performance of hydrogel contact lens by applying the surface patterning techniques.

## VIII. CURRENT LIMITATIONS, FUTURE CHALLENGES, AND CONCLUSIONS

### A. Limitations of the review paper

This review paper aims to provide a review of the current progress of topographical patterning on hydrogel materials, in particular, on the aspects of patterning technologies compatible with hydrogel fabrication, the impact of topographical patterning on surface energy, mechanical properties, and the subsequent influences on hydrogel–microbial and hydrogel–cell interactions. These changes in surface properties could affect the utilization of hydrogel in biomedical applications.

However, due to technological limitations, which will be further discussed in Sec. VIII B, important hydrogel properties, such as the changes in chemical moiety by topographical patterning, have been scarcely reported in the literature. Most of the studies that applied topographical patterning on hydrogel are interested in examining the changes in the interfacial energy changes. The most commonly used control unpatterned samples in most studies have been fabricated with the same methods. While a few studies reported the chemical characterization of the patterned hydrogel, thorough chemical moiety characterizations were not commonly reported. Therefore, this review paper also focused on changes in interfacial energy and hydrophobicity.

The topographical pattern design or optimal pattern for each different application has yet to be identified. The current approaches employed in topographical pattern design would be mainly biomimetic design, such as using lotus-leaf topography or performing a systematic screening of different patterns.

Moreover, we acknowledge that the hydrogel family also includes vast diversity of materials, and the hydrogel is a very versatile material with many potential applications. This review paper has only been focusing on the discussion on the examples of surface patterning of hydrogel for biomedical applications.

### B. Current and future challenges

Although topographically modified hydrogels have been demonstrated to show altered properties, some problems and challenges still exist in applying this technique for broader use.

As hydrogels can be used in different applications, the requirements they need to meet are also different. A hydrogel that can be ideally used in one area may not be suitable in another area. For example, an antibacterial hydrogel may not be suitable for cell culture design as the adhesion of cells could also be inhibited. Therefore, still a lot of work is necessary for each kind of hydrogel and each application.

Also, the relationship between topography features and their effects is still far from being well understood. There can be more than thousands of different patterns by varying their shape, height, spacing distance, and arrangements. However, most studies only selected one or several specific patterns for testing without showing the reason why those patterns were chosen. The exact mechanism of the pattern features and the changes they can make for hydrogels has not been thoroughly elucidated yet. This reduces the repeatability of a pattern to be used in various applications.

Surface characterization of patterns on hydrogel is another challenge. Common techniques that can be used to characterize micro- or nanoscale features, such as atomic force microscope (AFM), scanning electron microscope (SEM), and noncontact confocal base surface profiler, are challenging for hydrogels. These techniques were developed for the characterization of dry, hard, and/or refractive materials, while hydrogels are usually soft, hydrated, and transparent with low refractive index similar to air and water. Although AFM characterization can be performed in liquid chamber, specific setup and skilled operator would be needed. Additional processing steps, such as dehydration, freezing, and sputter coating, are necessary for hydrogel sample preparation for SEM and AFM characterization in air; however, these sample preparations may affect or even destroy the patterns. If the actual surface pattern on the hydrogel cannot be precisely detected, the analysis on how and why surface topography could alter surface properties will also be difficult.

Finally, while a pattern can modify properties of hydrogel, not all properties can be changed to the desired condition because material properties are coupled to and influenced by each other. To determine a suitable and successful material for a specific application, careful selection and optimization would be necessary to modify the surface without sacrificing other desirable properties. When topography is incorporated on a hydrogel, other properties, for example, surface hydrophobicity, may also be altered. For example, in the surface hydrophobicity altered by topography, the resulted changes in protein adsorption and the topography can affect cell response behaviors, making it more difficult to guarantee the effectiveness.

### C. Future prospective

Hydrogel is a promising biomaterial. The ability to modify the surface properties independently from the bulk properties could further enhance the use of hydrogel in biomedical applications. In this reviewer paper, we have discussed the applications of the topographically patterned hydrogel as tools in studying mechanobiology,^42,171,227^ wound healing and contact lens applications,^232,240^ and tissue engineering applications.^23,56,81,97,154^ However, hydrogels have also been developed as stimuli-responsive materials,^242^ materials for cell encapsulation,^243^ drug delivery vehicles,^244,245^ microfluidic devices,^246,247^ or materials for constructing biosensors.^248,249^ The incorporation of topographical patterning could further enhance the cell- or protein-interaction with the materials. The ability to change the interfacial energy could also be employed to develop hydrogel for medical adhesive,^250,251^ or in the development of soft robotics.^252,253^

In the post-pandemic era, surface properties for infection controls would be an essential aspect for further research. Nanopatterning and nanoparticles have already been demonstrated and used in anti-microbial applications.^254,255^ The application of topographical patterning could be used together with other infectious control methodology on medical devices.

### D. Conclusion

Compared with other biomaterials, hydrogels have shown outstanding performance, such as high hydrophilicity and biocompatibility. In order to further modify hydrogel properties for specific applications in different areas, different modifications were developed. Among them, surface topography modification provided new ideas on changing hydrogel surface properties. Several studies have successfully figured out the techniques that can be used to pattern hydrogels. Adding topographies onto hydrogel surfaces has been shown to affect the hydrophobicity, microbial adhesion, protein deposition, and cell behaviors on the hydrogel, making it a promising method to expand the applications of hydrogels. However, further systematic research will still be essential and necessary in understanding the relationship between topography features and their effects.

## Data Availability

Data sharing is not applicable to this article as no new data were created or analyzed in this study.
